# Ginsenoside Rb1 Ameliorates Diabetic Arterial Stiffening *via* AMPK Pathway

**DOI:** 10.3389/fphar.2021.753881

**Published:** 2021-10-12

**Authors:** Xinyu Zhang, Lei Wang, Rong Guo, Jie Xiao, Xiaoling Liu, Mei Dong, Xiaorong Luan, Xiaoping Ji, Huixia Lu

**Affiliations:** ^1^ The Key Laboratory of Cardiovascular Remodeling and Function Research, Chinese Ministry of Education, Chinese National Health Commission and Chinese Academy of Medical Sciences, The State and Shandong Province Joint Key Laboratory of Translational Cardiovascular Medicine, Department of Cardiology, Qilu Hospital, Cheeloo College of Medicine, Shandong University, Jinan, China; ^2^ Department of Cardiology, Ji’an Municipal Center People’s Hospital, Ji’an, China; ^3^ Dr. Gilbert Hung Ginseng Laboratory, Department of Biology, Faculty of Science, Hong Kong Baptist University, Hong Kong, Hong Kong, SAR China

**Keywords:** Rb1, arterial stiffening, AMPK, diabetes, ginsenoside

## Abstract

**Background and Purpose:** Macrovascular complication of diabetes mellitus, characterized by increased aortic stiffness, is a major cause leading to many adverse clinical outcomes. It has been reported that ginsenoside Rb1 (Rb1) can improve glucose tolerance, enhance insulin activity, and restore the impaired endothelial functions in animal models. The aim of this study was to explore whether Rb1 could alleviate the pathophysiological process of arterial stiffening in diabetes and its potential mechanisms.

**Experimental Approach:** Diabetes was induced in male C57BL/6 mice by administration of streptozotocin. These mice were randomly selected for treatment with Rb1 (10−60 mg/kg, i. p.) once daily for 8 weeks. Aortic stiffness was assessed using ultrasound and measurement of blood pressure and relaxant responses in the aortic rings. Mechanisms of Rb1 treatment were studied in MOVAS-1 VSMCs cultured in a high-glucose medium.

**Key Results:** Rb1 improved DM-induced arterial stiffening and the impaired aortic compliance and endothelium-dependent vasodilation. Rb1 ameliorated DM-induced aortic remodeling characterized by collagen deposition and elastic fibers disorder. MMP2, MMP9, and TGFβ1/Smad2/3 pathways were involved in this process. In addition, Rb1-mediated improvement of arterial stiffness was partly achieved via inhibiting oxidative stress in DM mice, involving regulating NADPH oxidase. Finally, Rb1 could blunt the inhibition effects of DM on AMPK phosphorylation.

**Conclusion and Implications:** Rb1 may represent a novel prevention strategy to alleviate collagen deposition and degradation to prevent diabetic macroangiopathy and diabetes-related complications.

## Introduction

Diabetes mellitus (DM) is one of the costliest and most burdensome chronic diseases worldwide. It has become a pandemic health disaster, especially among the elderly. In addition to the disease, consequent chronic vascular complications are a major cause of the increased morbidity and mortality of diabetic patients (Delbin and Trask, 2014). Several clinical trials have confirmed that intensive glycemic control in people with diabetes contributes to reducing the risk of microvascular (Holman et al., 2008). However, there was no evidence that it has advantages in terms of mortality or diabetic macroangiopathy (Zoungas et al., 2014). Macrovascular complications of DM, characterized by increased aortic stiffness, are also associated with hypertension, aging, insulin resistance, atherosclerosis, and hypertriglyceridemia (Mitchell et al., 2007; Dietrich et al., 2010; Payne et al., 2010; Stacey et al., 2010). Increased aortic stiffness independently predicts future cardiovascular disease, especially in women (Laurent et al., 2012; Ben-Shlomo et al., 2014). It leads to many adverse clinical outcomes, including impaired coronary perfusion and subsequent cardiovascular mortality.

**FIGURE 1:**
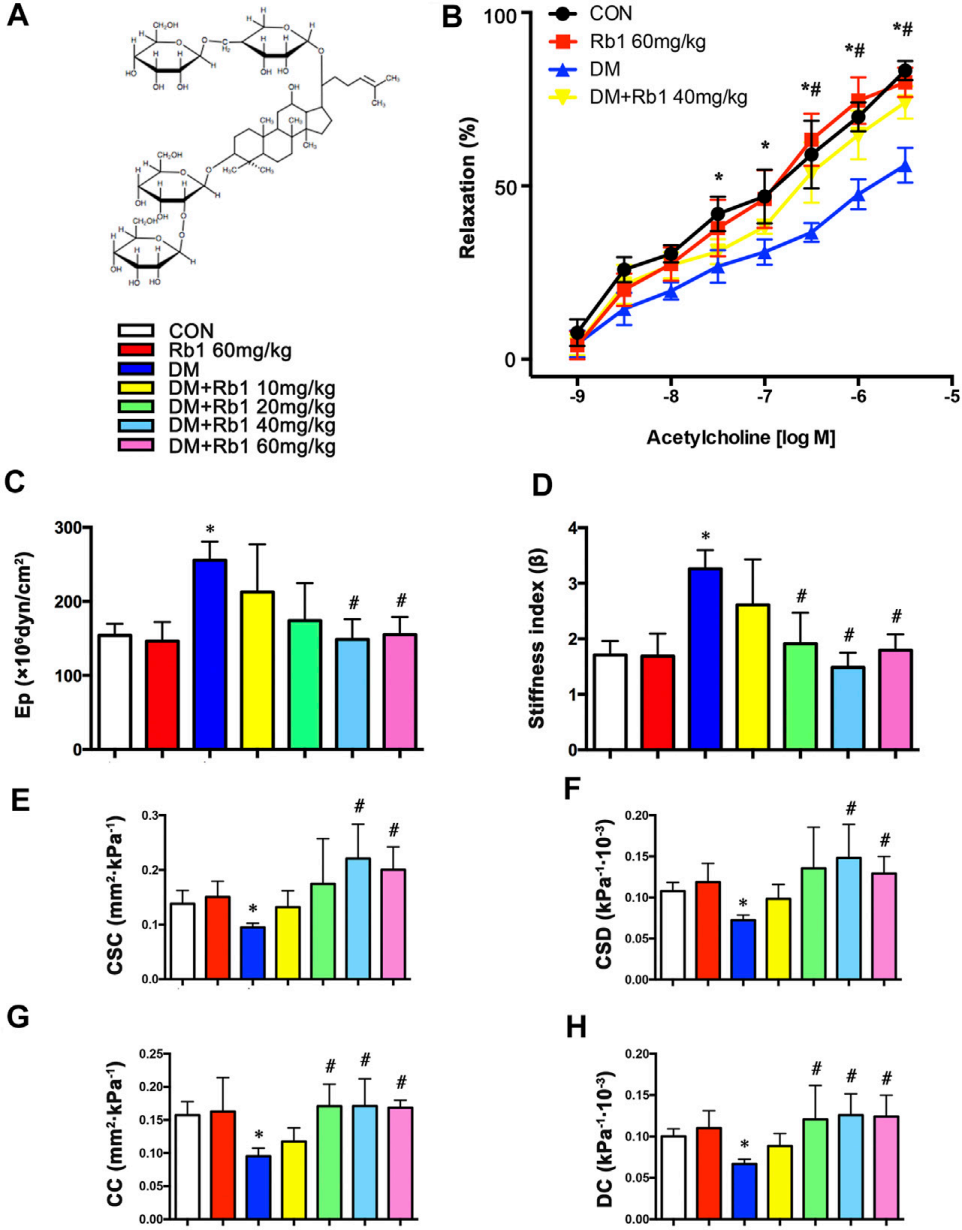
Rb1 improves aortic compliance and restores acetylcholine-induced endothelium-dependent vasorelaxation. **(A)** Chemical structure of ginsenoside Rb1. **(B)** After the addition of phenylephrine, cumulative doses of acetylcholine (1 × 10^–9^–1 × 10^–5.5^ M) were added to check the endothelial functions. **(C)** Peterson’s elastic modulus (Ep). **(D)** Arterial stiffness index (β). **(E)** Cross-sectional compliance (CSC), **(F)** cross-sectional distensibility (CSD), **(G)** compliance coefficient (CC), and **(H)** distensibility coefficient (DC). Data are mean ± SEM. n = 5–6, *p < 0.05 vs. Control; #*p* < 0.05 DM + Rb1 vs. DM.

As a major active component of ginseng, ginsenoside Rb1 (Rb1) (Figure 1A) (Cho et al., 2004) is the most frequently used and studied Chinese medicine and object. Gabriel Hoi-huen Chan et al. have demonstrated that ginseng extract exerted a protective effect in restoring normal endothelial functions in models with diabetes (Chan et al., 2013). Min Liu et al. have demonstrated that Rb1 reduced body weight, improved glucose tolerance, enhanced insulin action, and decreased the accumulation of cellular lipid in the livers of obese animals induced by high-fat diet (HFD) by activating the adenosine monophosphate (AMP)-activated protein kinase (AMPK) signaling pathway (Xiong et al., 2010; Shen et al., 2013). Interestingly, the effects of metformin, thiazolidinediones, and some other antidiabetic drugs are mediated through AMPK activation. Previous studies have supported the notion that AMPK working as a metabolic sensor of cellular adenosine triphosphate (ATP) levels is an important therapeutic target of aortic stiffness in cardiovascular diseases (CVDs) (Nagata et al., 2004; Gu et al., 2014; Lin et al., 2016).

These studies prompted us to hypothesize that Rb1 might alleviate the pathophysiological process of arterial stiffening in diabetes via the AMPK pathway. We used an animal model of type 1 diabetes to verify this hypothesis.

## Materials and Methods

### Cell Culture and Treatments

Dulbecco’s modified Eagle’s medium (DMEM) supplemented with 10% fetal bovine serum (FBS) and penicillin (100 U/ml) or streptomycin (100 μg/ml) maintained MOVAS-1 murine primary aortic vascular smooth muscle cells (VSMCs) (ATCC; Cat. no CRL-2797TM) at the temperature of 37°C with 5% CO_2_ atmosphere in a humid incubator. Upon reaching 60–70% confluence, cells were incubated with control medium (NC, 5.5 mmol/L) and serum-free DMEM overnight before treatment with high-glucose medium (HG, 30 mmol/L) and Rb1. VSMCs were stimulated with Rb1 (40 μM) 2 h before HG (30 mM) stimulation and cultured for an additional 48 h. For the HG + Rb1 + compound C (p-AMPK inhibitor) group, VSMCs were pretreated with compound C (10 μM) for 2 h before Rb1 treatment. Compound C was purchased from Selleck (Houston, Texas, the United States) and dissolved in dimethylsulfoxide (DMSO). Cells and supernatant were harvested simultaneously.

### Mice and Drug Treatment

This study followed the animal protocols approved by the Animal Care Committee of Shandong University and the Guide for the Care and Use of Laboratory Animals published by the National Institutes of Health. All mouse husbandry and experiments followed the Animal Management Rule of the Ministry of Health of the People’s Republic of China (Document No. 55, 2001). Male C57BL/6 mice (6–8 weeks, 25–28 g, Vital River Laboratories, Beijing, China) were classified into control and diabetes mellitus groups (CON and DM (n = 15 and 90)). As mentioned earlier, streptozocin (STZ) induced diabetes (Wang et al., 2014; Zhang et al., 2016). In brief, mice (n = 75 and 15) from DM and CON groups were randomly selected for treatment with Rb1 dissolved in normal saline and intraperitoneally (ip) once every day for 8 weeks in DM + Rb1 and CON + Rb1 groups. The dose range of Rb1 (10–60 mg/kg) was based on other experimental studies (Jiang et al., 2007; Zhao et al., 2010).

### Blood Pressure Measurement

As described previously, systolic and diastolic blood pressures (SBP and DBP) were measured using a noninvasive tail-cuff system (Softron BP-98A; Softron, Tokyo, Japan) (Kanda et al., 2005) and used for calculating pulse pressure (PP).

### Arterial Stiffness Assessment

As previously mentioned, the Vevo2100 imaging system (Visual Sonics, Toronto, Canada) was utilized to perform aortic ultrasonography (Zhang et al., 2016). Isoflurane (1% in O_2_) was inhaled by and anesthetized mice. Two-dimensional (2D), M-mode, and pulsed wave (PW) Doppler was used to obtain images. Three continuous cardiac cycles were averaged to get all measurements conducted by an operator. Minimum and maximum (end-diastolic, Dd; peak systolic, Ds) diameters were obtained from M-mode. 2D ultrasonography was applied to determine Peterson’s elastic modulus (Ep), arterial stiffness index (β), cross-sectional distensibility and compliance (CSD and CSC), and distensibility and compliance coefficients (DC and CC), which were estimated automatically by the following formulae (Pannier et al., 2002):

$$EP=\left(\frac{\Delta P}{\Delta D}\right)\times Dd=\left[\frac{Ps-Pd}{Ds-Dd}\right]\times Dd\left(\frac{10^{6}dym}{cm^{2}}\right)\;\;\beta =ln\frac{Ps/Pd}{\left(\left[Ds-Dd\right]/Dd\right)},$$

$$CSC=\frac{\Delta V/L}{\Delta P}=\frac{\mathrm{\Delta Α}}{\Delta P}=\frac{\pi \times \left(2Dd\times \Delta D\times \Delta D^{2}\right)}{\mathrm{4\Delta}P}\left(mm^{2}\cdot kPa^{-1}\right),$$

$$\begin{array}{l} CSD=\Delta A:\left[A\times \left(Ps-Pd\right)\right]=\pi \times \left[{\left(\frac{Ds}{2}\right)}^{2}-{\left(\frac{Dd}{2}\right)}^{2}\right]:\left[\pi \times {\left(\frac{Dd}{2}\right)}^{2}\times \left(Ps-Pd\right)\times 0.13332\right]\\ =\frac{2Dd\times \Delta D+Dd^{2}}{Dd^{2}\times \Delta P}\left(kPa^{-1}\cdot 10^{-3}\right), \end{array}$$

$$CC=\frac{2Dd\times \Delta D+Dd^{2}}{\mathrm{4\Delta}P}\left(mm^{2}\cdot kPa^{-1}\right),DC=\frac{\mathrm{2\Delta}d}{Dd\times \Delta P}\left(kPa^{-1}\cdot 10^{-3}\right)$$

where Ps and Pd are SBP and DBP, respectively; ∆P, ∆D, and ∆A represent the changes in BP, vascular diameter, and aortic cross-sectional lumen area, respectively; Ds and Dd stand for systolic and diastolic diameters, respectively; A refers to aortic cross-sectional lumen area.

### Measurement of Relaxant Responses in the Aortic Rings

Measurement was implemented as described previously (Chan et al., 2013). Briefly, mice were anesthetized, whose thoracic aortas were cut from the aortic arch to the diaphragm and immediately put into dishes containing Krebs buffer maintained at 4°C. Adipose tissues were cut off from the aortas before being cut into 3 mm segment rings. Then, the segments were mounted cautiously between two platinum hooks in 10 ml of organ baths, maintaining Krebs buffer at 37°C and continuously bubbled with 95% O_2_ to 5% CO_2_. After the 60 min equilibration of resting tension determined by normalization, each aortic ring was added with the cumulative doses of KCL (20–80 mM) to detect their activation. After the wash-out of KCL, the addition of one-dose phenylephrine at 1 × 10^–7^ M was performed until aortic rings maintained 50% of maximum tension. Endothelial functions were checked by adding the cumulative doses of acetylcholine (1 × 10^–9^–1 × 10^–5.5^ M). The plateau of responses was followed by the addition of all doses.

### Experimental Procedure

At last, mice were dissected and perfused with saline before being anesthetized with 1% pentobarbital sodium, which was then sacrificed, with thoracic aortas removed from the chest and rinsed with saline. A portion of the aorta (approximately 5 mm) underwent 72 h fixing in 4% paraformaldehyde, followed by the dehydration of tissues by ethanol and their embedment in paraffin, and the use of cross-sections (a thickness of 5 μm) for histological and morphometric analyses. Liquid nitrogen was used to freeze the rest of the aortas at once and store them at −80°C for subsequent molecular experiments.

### Histological and Morphometric Analyses

Sirius red and Verhoeff-Van Gieson (VVG) staining were used to stain the sections so as to shape and arrange collagen and elastin content, respectively. Sirius red slides are imaged using circularly polarized light showing newer, thinner collagen fibers as green and older, thicker fibers as red/orange. The VVG slides show both collagen (pink) and elastic fibers (black). The ratio of perivascular collagen area (PVCA) to the luminal area (LA) was utilized to represent perivascular collagen content for normalizing PVCA around vessels in a variety of sizes. Pictures were obtained under a microscope (BX52, Olympus, Tokyo, Japan) and analyzed using Image-Pro Plus 5.0 software (Media Cybernetics, US). The positive area and total tissue area of each image were obtained by analyzing the images. Collagen and elastic fibers content were quantified as a percentage of total tissue area. Histological and morphometric analyses were conducted by analyzing no less than three fields per section.

### Immunohistochemical and Immunofluorescence Staining

Regarding immunohistochemistry, 0.05 M sodium citrate buffer (a pH value of 6.0) was applied to perform heat-mediated antigen retrieval after the rehydration of tissue sections (5 μm). Three percent of hydrogen peroxide and bovine serum albumin were used to prevent endogenous peroxidase activity and non-specific staining, respectively. Primary antibodies against collagens Ι and Ⅲ, 3-ni0trotyrosine (Abcam, Cambridge, the United Kingdom), and fibronectin (Proteintech Group, Chicago, Illinois (IL), the United States) were added and incubated at 4°C in a humidified box for one night. A secondary antibody (Beijing Zhong Shan-Golden Bridge Biological Technology Co., Ltd. China) was applied to incubate the sections washed with phosphate-buffered saline at 37°C for half an hour for immunohistochemical staining. Diaminobenzidine (DAB) solution (Beijing Zhong Shan-Golden Bridge Biological Technology Co., Ltd. China) was used to incubate the sections washed with phosphate-buffered saline. Hematoxylin was used to counterstain nuclei. For immunofluorescence staining, the incubation of the sections was performed by fluorescein isothiocyanate (FITC)-conjugated antibodies (a ratio of 1:50, ZSGB-BIO, Beijing, China). 4′6-Diamidine-2-phenylindole dihydrochloride (DAPI) (a ratio of 1:200, Roche, Germany) was used to stain nuclei. The observation of tissue sections was conducted using a FV 1000 SPD laser-scanning confocal microscope (Olympus, Japan). The software Image-Pro Plus 5.0 was used to analyze the obtained images. The area and IOD of each image were obtained by analyzing the images, and the mean intensity can be calculated by IOD/area. The analysis of no less than three fields per section was carried out.

### Assessment of Intracellular ROS Levels

The measurement of reactive oxygen species (ROS) production in VSMC was conducted by 2′,7′-dichlorodihydro-fluorescein diacetate (DCFH-DA; Biotime), Amplex Red (Molecular Probes, Invitrogen), and dihydroethidium (DHE; Biotime) according to the instructions of manufacturers.

### Western Blot Analysis

After separation by 8–10% sodium dodecyl sulfate (SDS)-polyacrylamide gel electrophoresis, proteins were moved to polyvinylidene difluoride membranes (0.22 and 0.45  μm, Millipore, Billerica, Massachusetts (MA), the United States). Overnight incubation was performed using antibodies against phospho-AMPK (Thr172), AMPK, collagens Ι and Ⅲ (Proteintech Group, Chicago, IL, the United States), phospho-Smad2 and Smad3, NOX1, NOX4 (Abcam, Cambridge, the United Kingdom), Smad2/3 (Millipore, Billerica, MA, the United States), glyceraldehyde-3-phosphate dehydrogenase (GAPDH) and β-actin (Beijing Zhong Shan-Golden Bridge Biological Technology Co., Ltd. China), matrix metalloprotein (MMP-9), and transforming growth factor-β1 (TGFβ1). The secondary antibody conjugated to horseradish peroxidase (Beijing Zhong Shan-Golden Bridge Biological Technology Co., Ltd. China) was used for the 1.5 h incubation of the membranes washed with Western washing buffer (TBS-T) at ambient temperature. The ECL kit (Millipore, Billerica, MA, the United States) was used to visualize immunoreactive bands, and the ChemiDoc™ Touch Imaging System (Bio-Rad Laboratories, Hercules, California, the United States) was utilized to obtain pictures.

### Real-Time Quantitative Reverse-Transcriptase PCR (RT-qPCR)

A ribonucleic acid (RNA) extraction kit (Qiagen) was employed to prepare total cellular RNA. The following primers were used to perform real-time reverse-transcriptase quantitative polymerase chain reaction (RT-qPCR). For the analysis of vascular NOX1 messenger RNA (mRNA), the primer sequences are as follows: forward and reverse: 5′GCT​CCA​GAC​CTC​CAT​TTG​ACA3′ and 5′AAG​GCC​AAG​GCA​GTT​CCG​AG3′, respectively. For the analysis of vascular NOX2 mRNA, the primer sequences are as follows: forward and reverse: 5′CAC​TTC​ACA​CGG​CCA​TTC​AC3′ and 5′ACC​GAG​TCA​CAG​CCA​CAT​AC3′, respectively. For the analysis of vascular NOX4 mRNA, the primer sequences are as follows: forward and reverse: 5′ATG​TGG​GCC​TAG​GAT​TGT​GT3′ and 5′CCT​GCT​AGG​GAC​CTT​CTG​TG3′, respectively. For the analysis of GAPDH mRNA the primer sequences are as follows: forward and reverse: 5′GCT​GTG​ATC​CTG​AGC​TCC​GAG​AC3′ and 5′CAT​GTG​GGC​CAG​GTC​CAC​CAC3′, respectively. For the analysis of VSMC NOX1 mRNA, the primer sequences are as follows: forward and reverse: 5′GGT​TGG​GGC​TGA​ACA​TTT​TTC3′ and 5′TCG​ACA​CAC​AGG​AAT​CAG​GAT3′, respectively. For the analysis of VSMC NOX4 mRNA, the primer sequences are as follows: forward and reverse: 5′GAA​GGG​GTT​AAA​CAC​CTC​TGC3′ and 5′ATG​CTC​TGC​TTA​AAC​ACA​ATC​CT3′, respectively. Synergy brands (SYBR) green was used as fluorescence dye to carry out reactions on a real-time PCR system (LightCycler 96, Roche). Experiments were conducted twice. The 2^-△△CT^ method was adopted in relative expression analysis.

### Statistical Analysis

Data were reported to be the mean ± standard error mean (SEM). First, the homogeneity of variance and Kolmogorov–Smirnov tests were performed. Then, the one-way analysis of variance (ANOVA) was conducted to analyze multiple groups, and *post hoc* individual comparisons were made. Finally, the least significant difference (LSD) test was performed to compare the means of every group and other columns in the case of homogeneous variance, and *p*-value was obtained by performing Dunnett’s T3 test in the case of inhomogeneous variance. Differences were considered statistically significant at *p* < 0.05. Statistical Product and Service Solutions (SPSS) v20.0 (SPSS Inc., Chicago, IL, the United States) was used in all statistical analyses.

## Results

### Characteristics of the Mice at the End of Experiments

At baseline, these groups showed no difference in BP, blood glucose, and body weight. In order to evaluate the relationship between Rb1 and body weight, parameters of mice, including blood glucose and BP, were measured after an 8-week Rb1 treatment. As shown in Table 1, the DM group had lower DBP and higher PP and PP/MBP compared with the CON one but saw a drop after Rb1 treatment (40 and 60 mg/kg). Body weight showed no significant differences after Rb1 treatment (Table 1). Glucose levels presented a decreasing trend in the high-dose group compared with those in the DM one, whereas both groups were not statistically different (*p* = 0.537).

**TABLE 1:** Characteristics of the mice at the end of experiment.

	Control	Rb1	DM	DM + Rb1 (10 mg/kg)	DM + Rb1 (20 mg/kg)	DM + Rb1 (40 mg/kg)	DM + Rb1 (60 mg/kg)
**HR (bpm)**	626.143 ± 17.856	618.6 ± 11.717	610.6 ± 29.828	647.421 ± 16.831	591.529 ± 20.268	613.5 ± 18.853	612.632 ± 7.626
**BW (g)**	30.2 ± 0.961	29.575 ± 0.630	23.75 ± 1.386^*^	25.675 ± 0.669^*^	25.462 ± 0.662^*^	24.628 ± 0.434^*^	25.65 ± 0.715^*^
**SBP** **(mmHg)**	106.6 ± 5.653	102.3333 ± 6.386	102.25 ± 3.240	100.8 ± 7.276	106.25 ± 3.966	113.857 ± 4.295^#^	105.889 ± 1.867
**DBP (mmHg)**	78 ± 4.868	79.333 ± 9.333	60.438 ± 2.871^*^	66.4 ± 3.027	77 ± 4.916^#^	85.5 ± 2.754^#^	80.778 ± 3.833^#^
**PP (mmHg)**	20.75 ± 2.955	23 ± 4.359	41.813 ± 4.078^*^	34.4 ± 5.609	29.25 ± 7.825	25.429 ± 3.741^#^	25.111 ± 2.816^#^
**PP/MBP**	0.3325 ± 0.1021	0.2751 ± 0.0668	0.4826 ± 0.0249^*^	0.4367 ± 0.0563	0.3439 ± 0.0992	0.2752 ± 0.0432^#^	0.3148 ± 0.0389^#^
**GLU (mmol/L)**	8.488 ± 0.910	8.486 ± 1.063	25.263 ± 2.045^*^	24.362 ± 2.018^*^	22.233 ± 1.810^*^	23.222 ± 2.960^*^	21.500 ± 1.877^*^

Data are mean ± SEM, n = 5–8 per group. HR, heart rate; BW, body weight; SBP, systolic blood pressure; DBP, diastolic blood pressure; PP, pulse pressure; MBP, mean blood pressure; GLU glucose.* *p* < 0.05 vs. Control; # *p* < 0.05 DM + +Rb1 vs. DM

### Recovery of Endothelial Function and Aortic Compliance Following Rb1 Treatment

In the present study, we examined endothelial functions and aortic compliance. In the DM group, endothelium-dependent vasodilatory responses to acetylcholine were decreased compared with those in the CON group (E_max_ = 56.0 ± 8.8% vs. E_max_ = 83.3 ± 4.7%), indicating that DM has induced endothelial dysfunction. These defects were improved by Rb1 treatment (E_max_ = 74.1 ± 8.1%) (Figure 1B).

Reflecting worse aortic compliance, the increase of Ep and arterial stiffness index in DM mice (Figures 1C,D) was reverted after treatment with Rb1 as well. In contrast, Rb1-treated mice showed an increase in CSD, CSC, DC, and CC compared with DM ones (Figures 1E–H). It was observed that Rb1 had a maximum effect at 40 mg/kg, which was thus chosen for further research.

### Rb1 Alleviated DM-Induced Aortic Remodeling

**FIGURE 2:**
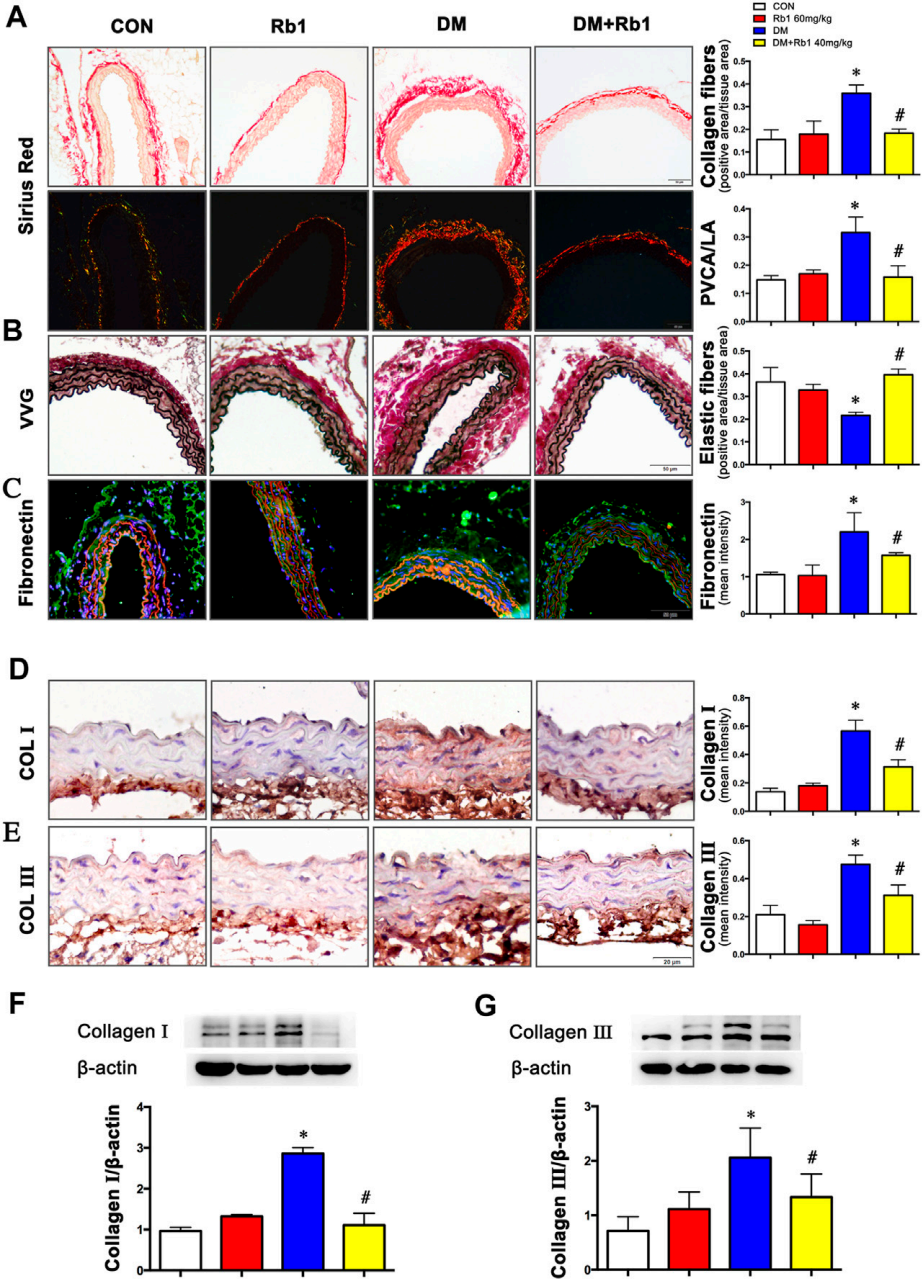
Rb1 reduces DM-induced aortic remodeling **(A)** Sirius red staining (bright field and dark field) (scale bar 50 μm). Collagen fibers were stained bright red. Semiquantitative analysis of collagen fibers. Perivascular collagen content, shown as the perivascular collagen area/luminal area (PVCA/LA) ratio. **(B)** Elastic fibers shown by Verhoeff-Van Gieson staining (scale bar 50 μm). Elastic fibers are black, VSMCs are light red, and collagen fibers are pink. Semiquantitative analysis of elastic fibers. **(C)** Fibronectin accumulation (green: fibronectin, red: elastic lamina, blue: nuclei, scale bar 50 μm). Semiquantitative analysis of fibronectin expression. Representative immunohistochemical staining **(D)**, Western blot bands **(F)**, and semiquantitative analysis of collagen I expression (scale bar 20 μm). Representative immunohistochemical staining **(E)**, Western blot bands **(G)**, and semiquantitative analysis of collagen Ⅲ expression (scale bar 20 μm). Data are mean ± SEM. n = 5–6, *p < 0.05 vs. Control; #*p* < 0.05 DM + Rb1 vs. DM.

Collagen fibers were stained bright red, shown by Sirius red staining. Evaluated by collagen fiber area and PVCA/LA ratio, adventitial collagen accumulation showed an enhancement in DM mice relative to CON ones and was prevented by Rb1 treatment (Figures 2A,B). Additionally, diabetic mice exhibited higher values of PVCA/LA compared with CON ones, whereas enhanced values were reversed after treatment with Rb1 (Figure 2A). Elastic fibers were black shown by VVG staining, which suggested that focal irregularities and insufficient normal wavy contraction in the arrangement of elastic fibers in DM mice were not found in CON ones. Rb1 treatment contributed to improvement in the elastic lamina, with fewer inordinate patterns in the CON group than in the DM one (Figure 2B). Fibronectin in the adventitia was observed in all groups, whose accumulation was higher in the adventitia of the DM group than that of the CON one but regressed by Rb1 (Figure 2C). Moreover, the immunohistochemical detection of collagens I and III demonstrated that the aorta of the DM group had stronger immunostaining than that of the CON one (Figures 2D,E). Similarly, the protein expressions of collagens I and III exhibited higher levels in DM mice than in CON ones (Figures 2F,G). The accumulation of collagens I and III in diabetes was regressed after treatment with Rb1 (Figures 2D–G).

### AMPK Involved in the Effects of Rb1 on Collagen Accumulation and TGFβ1-Smad2/3 Signaling Pathway

**FIGURE 3:**
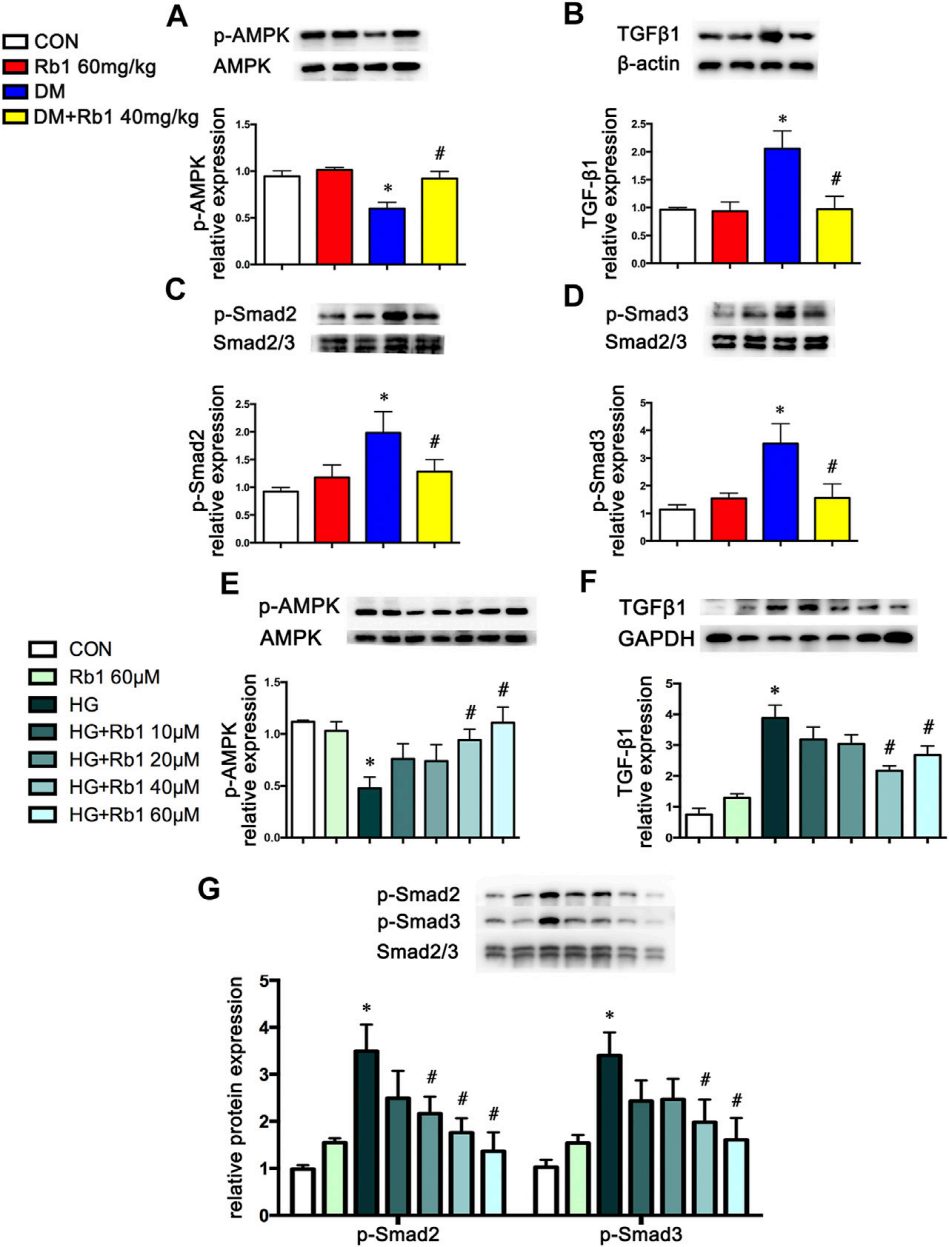
AMPK pathway is involved in the effect of Rb1 on DM- and HG-mediated TGFβ1, Smad2/3, and collagen expression. Representative Western blot bands and semiquantitative analysis of **(A)** phosphorylated AMPK (p-AMPK), **(B)** TGFβ1, **(C)** phosphorylated Smad2 (p-Smad2), and **(D)** phosphorylated Smad3 (p-Smad3) in aorta extracts. **(E–G)** VSMCs were treated with Rb1 at doses of 10, 20, 40, and 60 μM in a high-glucose medium. Representative Western blot bands and semiquantitative analysis of phosphorylated AMPK (p-AMPK), TGFβ1, phosphorylated Smad2 (p-Smad2), and phosphorylated Smad3 (p-Smad3). Semiquantitative analysis of above proteins expressions. Data are mean ± SEM. n = 5–6, *p < 0.05 vs. Control; #*p* < 0.05 DM + Rb1 vs. DM and HG + Rb1 vs. HG.

Aorta extracts from DM mice saw a decrease in AMPK phosphorylation and an increase in TGF β1 and phospho-Smad2/3 expressions compared with those from control ones (Figures 3A–D). After an 8-week Rb1 treatment, the inhibition of AMPK phosphorylation was reduced in Rb1-treated DM mice compared with the DM ones (Figure 3A). Meanwhile, TGFβ1 and phospho-Smad2/3 were suppressed in expression level (Figures 3B–D).

**FIGURE 4:**
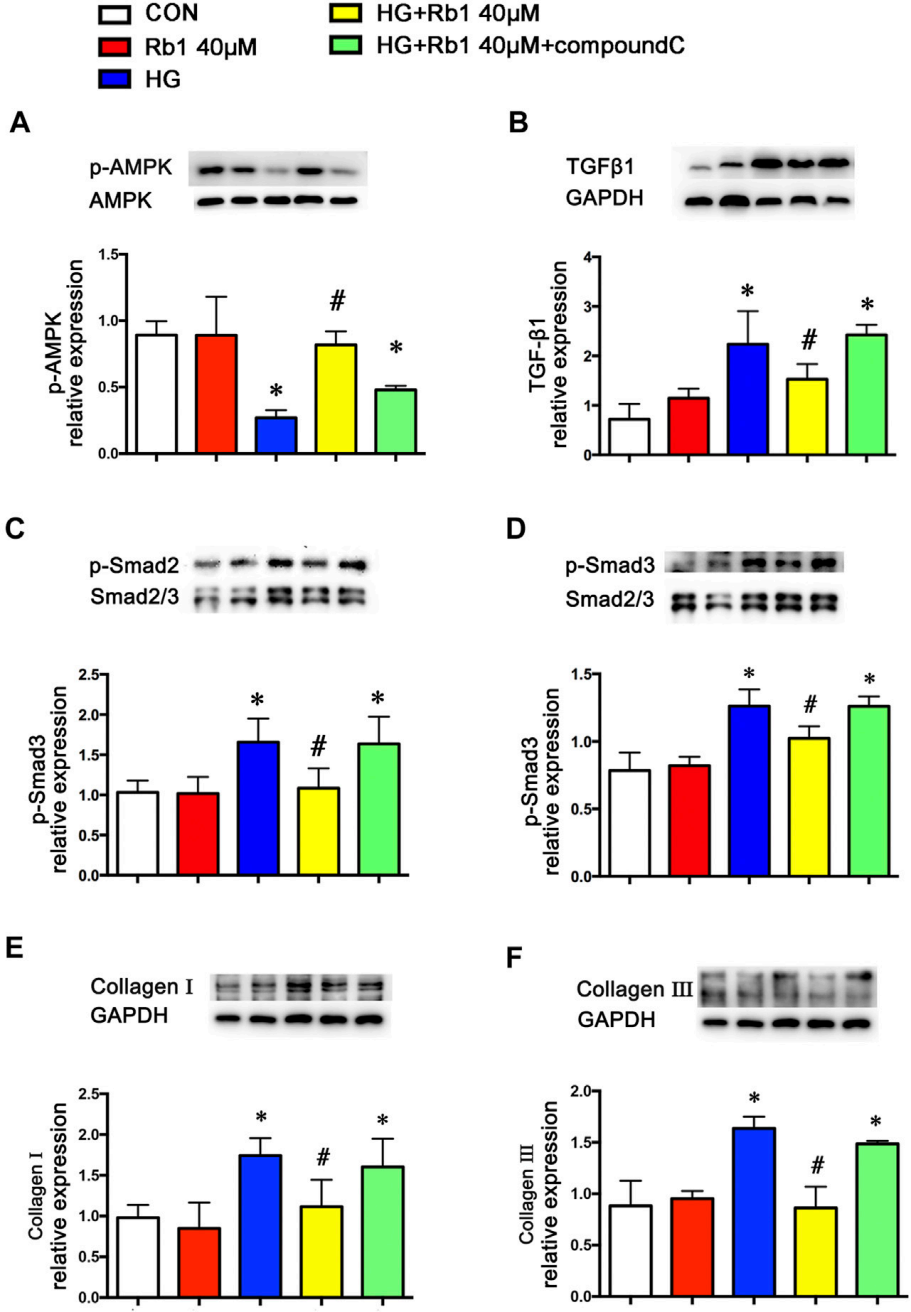
Rb1-mediated reduction of TGFβ1 and phospho-Smad2/3 and collagen accumulation are partly abolished by treatment with compound C, an inhibitor of AMPK. VSMCs were treated with Rb1 at doses of 40 μM in a high-glucose medium and pretreated with compound C. Representative Western blot bands and semiquantitative analysis of **(A)** phosphorylated AMPK (p-AMPK), **(B)** TGFβ1, and **(C)** phosphorylated Smad2 (p-Smad2). **(D)** Phosphorylated Smad3 (p-Smad3). **(E)** Collagen I and **(F)** collagen III. Data are mean ± SEM. n = 5–6, *p < 0.05 vs. Control; #*p* < 0.05 HG + Rb1 vs. HG.

To clarify the potential role of Rb1 treatment in this signaling pathway *in vitro*, VSMCs were pretreated with the concentration gradient of Rb1 (from 10 to 60 μM) 2 h before high-glucose (30 mM) (HG) stimulation and were cultured for an additional 48 h. We detected the levels of phospho-AMPK, TGFβ1, and phospho-Smad2/3 and selected 40 μM as the Rb1 treatment concentration (Figures 3E–G). Then, VSMCs were stimulated with Rb1 (40 μM) 2 h before high-glucose (30 mM) (HG) stimulation and were cultured for an additional 48 h. For the HG + Rb1+compound C group, VSMCs were pretreated with compound C for 2 h before the Rb1 treatment. The results exhibited that Rb1-mediated increase of phospho-AMPK, reduction of TGFβ1 and phospho-Smad2/3 in DM mice, and inhibition of collagenⅠand collagenⅢ accumulation were partly abolished by treatment with compound C, an inhibitor of AMPK (Figures 4A–F).

### Rb1 Reduced the Collagen Deposition, MMP-2, and MMP-9 Expression in VSMC

**FIGURE 5:**
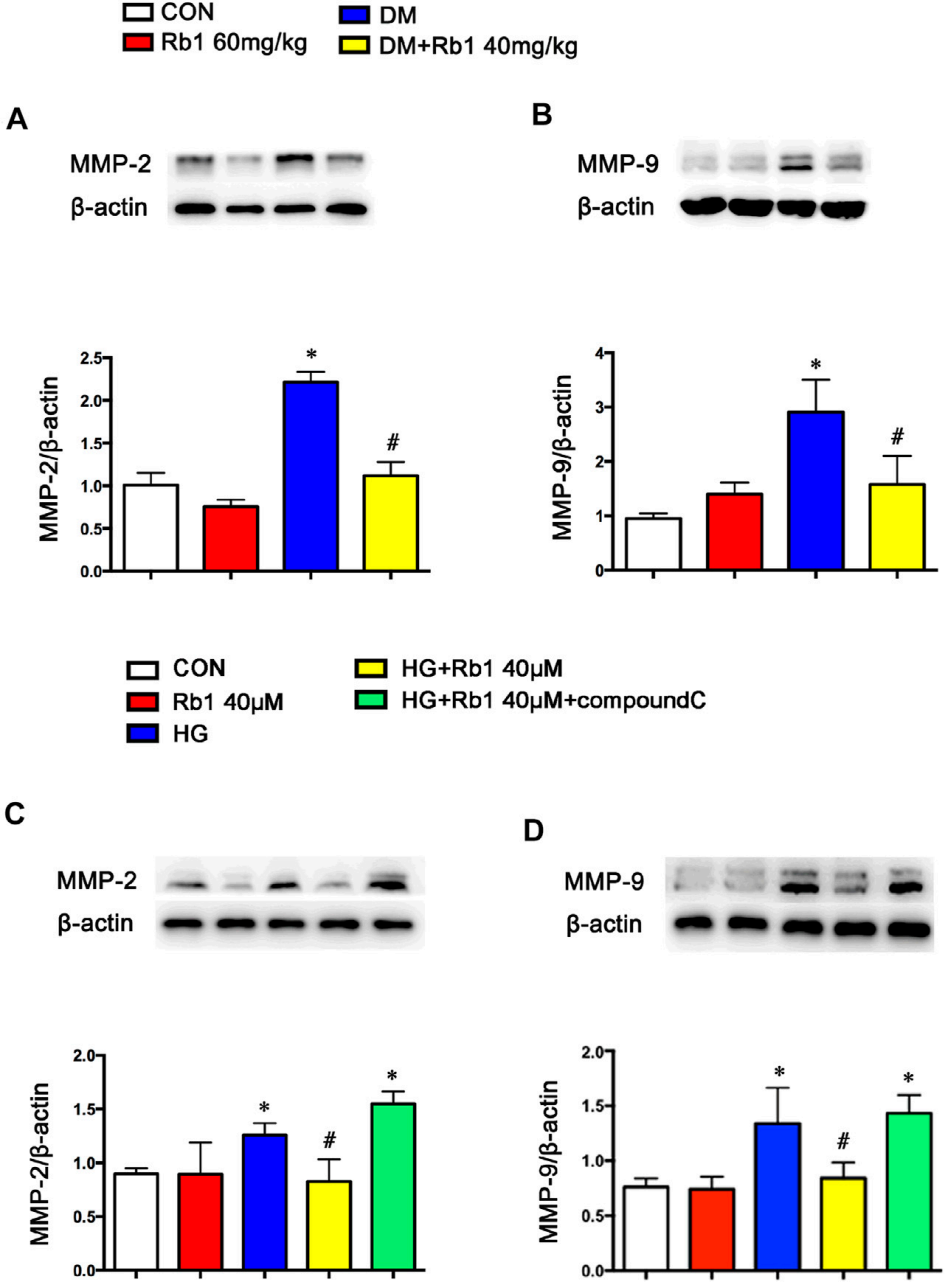
Rb1 suppresses MMP-2 and MMP-9 expression. Representative Western blot bands and semiquantitative analysis of **(A)** MMP-2 and **(B)** MMP-9 protein expression in aorta. Representative Western blot bands and semiquantitative analysis of **(C)** MMP-2 and **(D)** MMP-9 protein expression in VSMCs. Data are mean ± SEM. n = 5–6, *p < 0.05 vs. Control; #*p* < 0.05 DM + Rb1 vs. DM and HG + Rb1 vs. HG.

In addition to the TGFβ1-phospho-Smad2/3 pathway involved in vascular remodeling, we also detected MMPs expression and activity in diabetes and HG-treated VSMC. The results showed that the levels of MMP-2 and MMP-9 were increased (Figures 5A,B). However, the above alterations were partly reversed by Rb1. Meanwhile, Rb1 treatment (40 μM) inhibited the protein expression of MMP-2 and MMP-9 compared with high glucose (HG) without Rb1 treatment (Figures 5C,D). These effects were eliminated by treatment with compound C, an inhibitor of AMPK. Thus, Rb1 suppressed HG-induced collagen deposition and MMPs expression *via* the AMPK pathway.

### Rb1 Improved DM-Induced Oxidative Stress

**FIGURE 6:**
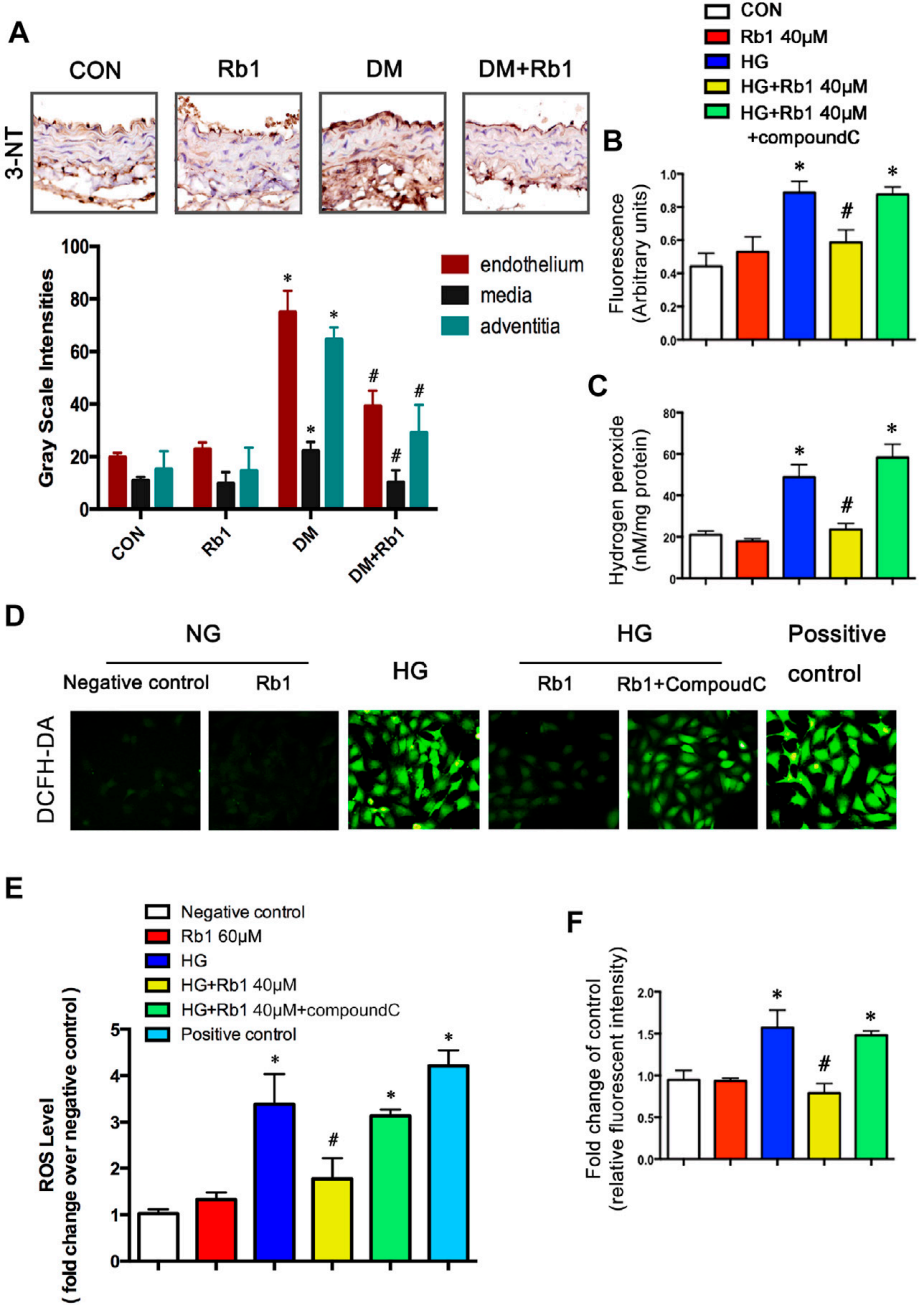
Rb1 improves DM and HG-induced oxidative stress. **(A)** Immunohistochemical staining and semiquantitative analysis of 3-nitrotyrosine (3-NT). ROS was assessed by three different methods: **(B)** DCFH-DA, **(C)** Amplex Red, and **(F)** DHE in VSMCs **(D)** ROS levels. in each group. Original magnification × 200. **(E)** Semiquantitative analysis of ROS levels in each group. The positive control is the active oxygen donor from the DCFH-DA kit, which contains H_2_O_2_. Data are mean ± SEM. n = 5–6, *p < 0.05 vs. Control; #*p* < 0.05 DM + Rb1 vs. DM and HG + Rb1 vs. HG.

To gain further insights into the potential protective mechanism of Rb1 in aortic remodeling, we assessed oxidative stress using 3-NT staining, an oxidative stress-induced lipid peroxidation marker. It was demonstrated that 3-NT staining was more evident in diabetic mice than in controls (Figure 6A). Staining was most intense in the endothelium, which is less in the adventitia and relatively minimal in the medial layer. Rb1 treatment prevented 3-NT accumulation in the endothelium and adventitia markedly. To further confirm whether Rb1 could decrease the production of ROS *in vitro*. ROS was assessed by three different methods, DCFH-DA, Amplex Red, and DHE in VSMCs (Figures 6B–F). Cells pretreated with Rb1 or compound C were exposed to high glucose (HG) for 48 h. Rb1 attenuated the HG-induced ROS level in cells. The protective effect of Rb1 was eliminated by treatment with compound C (Figures 6B–F), indicating that the inhibitory effect of Rb1 on ROS was AMPK-dependent.

### NOX Isoforms Involved in Effects of Rb1 on DM-Induced Oxidative Stress

**FIGURE 7:**
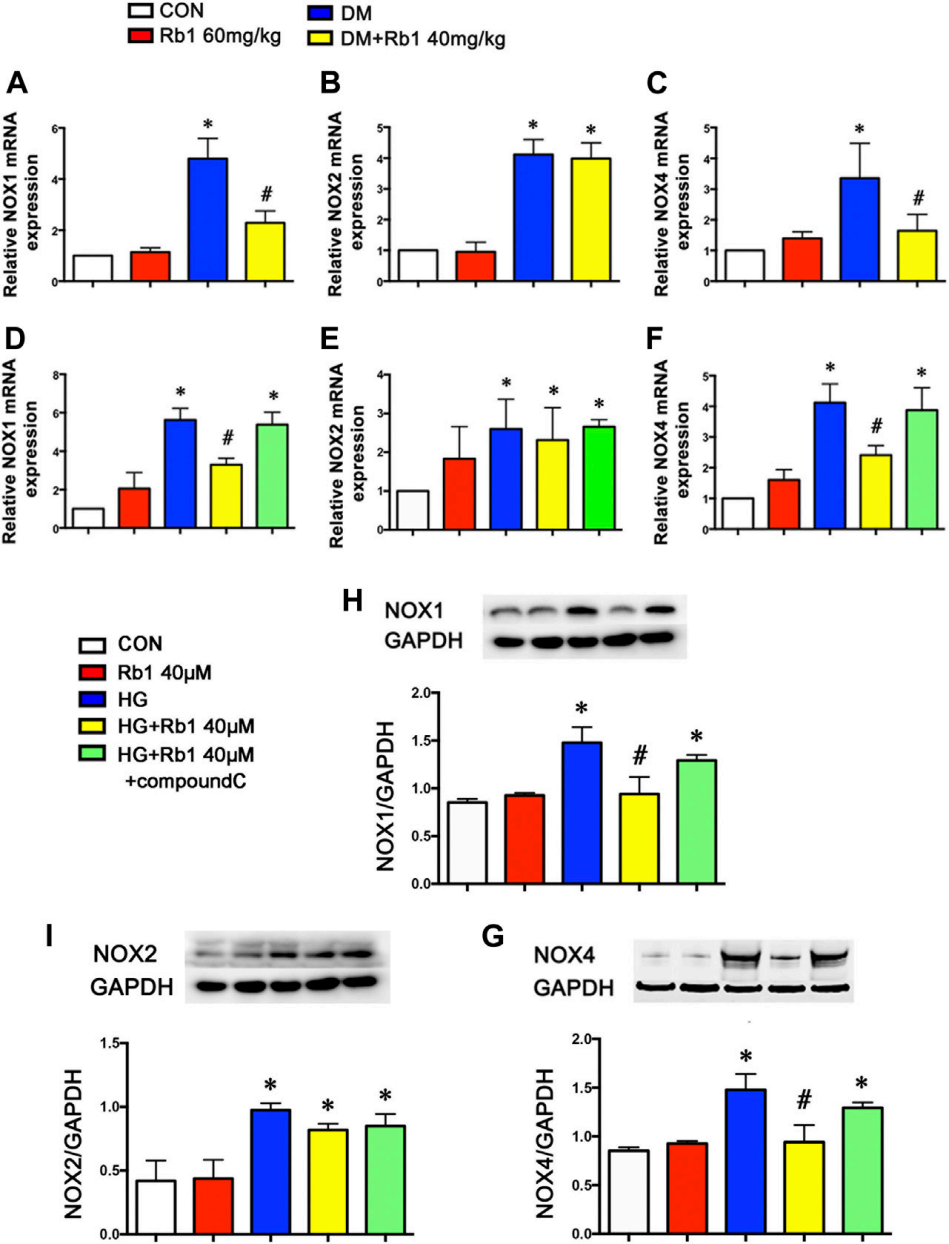
NOX isoforms involved in the effects of Rb1 on DM-induced oxidative stress. Quantitative analysis of **(A)** NOX1 **(B)** NOX2 and **(C)** NOX4 mRNA expression in the aorta. Quantitative analysis of **(D)** NOX1 **(E)** NOX2 and **(F)** NOX4 mRNA expression in VSMCs. Representative Western blot bands and semiquantitative analysis of **(H)** NOX1 **(I)** NOX2 and **(G)** NOX4 protein expression in VSMCs. Data are mean ± SEM. n = 5–6, *p < 0.05 vs. Control; #*p* < 0.05 DM + Rb1 vs. DM and HG + Rb1 vs. HG.

To clarify the potential effect of Rb1 treatment on the inhibition of oxidative stress, the mRNA levels of NOX1, NOX2, NOX4, and other NOX isoforms in aorta extracts were detected, indicating the inhibiting effect of Rb1 treatment on the mRNA expression levels of NOX1 and NOX4, which exhibited a rise in DM mice (Figures 7A–C). However, the DM group was not significantly different from the DM + Rb1 one in NOX2 (Figure 7B). Furthermore, the mRNA levels of NOX1, NOX2, and NOX4 in VSMCs were detected. The results showed that Rb1 had the same effect as aorta extracts (Figures 7D–F), and the changes of NOX1, NOX2, and NOX4 were confirmed in protein expression level (Figures 7G–I). In addition, these effects on the inhibition of NOX1 and NOX4 were partly eliminated by treatment with compound C, an inhibitor of AMPK.

## Discussion

In the present study, we found that Rb1 could alleviate arterial stiffness by reducing aortic remodeling. The beneficial effects of Rb1 on vascular stiffness were achieved by suppressing oxidative stress and inhibiting the expression of collagenⅠ, collagen Ⅲ, MMPs, and TGFβ1/Smad2/3, which were, at least partially, AMPK-dependent.

Rb1 is a major active component of *Panax ginseng*, whose protective action against a few CVDs (Bai et al., 2018; Zhou et al., 2019), including abdominal aortic aneurysm (Zhang et al., 2015), hypertension-induced carotid arterial remodeling (Lin et al., 2015), myocardial ischemia/reperfusion injury (Wu et al., 2011; Xia et al., 2011), and hypertrophy (Kanda et al., 2005; Jiang et al., 2007), has been recently proved by *in vivo* and *in vitro* studies. Previous research discovered that Rb1 led to a decline in the accumulation of lipid and the area of atherosclerotic plaques through the skew of macrophages to the M2 phenotype and the improvement of lipid metabolism and autophagy in macrophage foam cells (Qiao et al., 2017; Zhang et al., 2018). Nevertheless, the effect of Rb1 on vascular diseases under hyperglycemia is unclear, whose contributing molecular mechanisms remain to be elucidated. This study found that treatment with Rb1 could alleviate DM-induced arterial stiffness.

As a key pathway linking diabetes to CVDs, arterial stiffness can decrease diastolic pressure and increase PP. Philips, J C et al. have demonstrated that PP increased and concomitantly decreased in DBP according to T1DM duration, in agreement with accelerated arterial stiffening due to chronic hyperglycemia. They have confirmed the validity of using the index PP/MBP previously proposed as a surrogate marker of arterial stiffness (Philips et al., 2009). It is a complex phenomenon that arises from the qualitative and quantitative variations in arterial wall components, giving rise to the redistribution of mechanical loads towards elastic materials, endothelial dysfunction, increased smooth muscle tone, the phenotypic modulation of adventitial fibroblasts to myofibroblasts, and chronic inflammation (Zhou et al., 2012). Research has shown that Rb1 treatment could decrease PP and PP/MBP, restore DBP, endothelial function, and aortic compliance, and suppress aortic remodeling. Endothelial-independent relaxation (e.g., SNP-induced relaxation) should be analyzed to confirm the possible effects of Rb1 on smooth muscle cells in further study. Rb1 treatment failed to decrease glucose levels. Based on previous studies, no consensus reports evaluated the effect of Rb1 on serum glucose. In this study, glucose levels showed a decreasing trend in the high-dose group compared with those in the DM one, whereas both groups were not statistically different (*p* = 0.537). Rb1 protected arteries from stiffening, which was independent of decreased glucose levels.

Previous studies have supported the notion that AMPK was an important therapeutic target of diabetes (Lin et al., 2016; Luo et al., 2016), including DM-induced macrovascular complications (Gu et al., 2014; Nagata et al., 2004). AMPK played a key role in protecting vascular dysfunction from hyperglycemia involving reversing oxidant damage (Sambuceti et al., 2009), reducing inflammation (Ha et al., 2014), and attenuating endothelial dysfunction (Tang et al., 2016). Of interest, multiple molecular mechanisms of Rb1 treatment have been proposed, including reduction of oxidative stress, apoptosis, and protein synthesis, *via* AMPK-dependent pathway and some other pathways (Cho et al., 2004; Zhao et al., 2010; Xia et al., 2011; Shen et al., 2013; Zhang et al., 2015). In our study, we found that Rb1 could reduce the suppression of AMPK caused by hyperglycemia. Diabetes is accompanied by oxidative stress characterized by elevated ROS levels in the cardiovascular system (Jay et al., 2006). We found oxidative stress in aortic sections from diabetic mice and abundant ROS production in VSMCs, consistent with other reports (San Martín et al., 2007).

The anti-oxidative stress mechanisms of Rb1 may involve both direct ROS scavenging (Lü et al., 2012) and indirect signaling effects. Recent studies have demonstrated that activating AMPK contributed to reversing oxidant damage (Sambuceti et al., 2009) partly by reducing ROS generation and increasing nitric oxide (NO) production (An et al., 2016). The NADPH oxidases protein family was a major source of ROS in vascular cells (Brown and Griendling, 2009; Lassègue and Griendling, 2010; Amanso and Griendling, 2012). Our findings have supported that Rb1 treatment inhibited DM-induced overexpression of NOX1 and NOX4, but not NOX2. These benefits in suppressing NADPH oxidase and ROS production were partly eliminated by treatment with compound C, an inhibitor of AMPK. It seemed that Rb1 treatment took part in inhibiting ROS production, at least partially, via the AMPK pathway. As for why Rb1 did not suppress NOX2, the relative study needs to be performed in the future.

Previous studies have demonstrated that the activation of matrix metalloproteinase (MMP)-2/9 was strongly correlated with the disorganization, stiffness, and calcification of elastic fibers and the dysfunction of vasomotion in the arterial vasculature (Longo et al., 2002; Yasmin et al., 2005; Chung et al., 2009). The lack of elastin fibers or collagen deposition in the arterial wall resulted in aortic remodeling and increased stiffness (Sangartit et al., 2014; Herrmann et al., 2015; Li et al., 2015). It was found that DM mice exhibited increased 3-nitrotyrosine (NT) staining, MMP2/9 expressions and perivascular fibrosis/lumen area, disorganized elastic, and collagen fibers. Besides, the increased expressions of collagens I and III indicated an increase in the deposition of collagen in the DM group. Concomitant Rb1 treatment prevented the above-mentioned changes and retained the normal morphology of aortic specimens, which confirmed the anti-arterial stiffness effect of Rb1.

Another important factor regulating collagen production in aortic remodeling is the TGFβ1/Smad2/3 pathway, which is closely related to oxidative stress. The data of this study supported that TGF signaling got involved in the production of HG-induced collagens and the accumulation of extracellular matrices, which are in line with previous reports (Kubota et al., 2003; Ha et al., 2016). Cytoplasmic signals are transmitted into the intracellular domain by TGF-β via its type I and II receptors. After direct phosphorylation by the TGF-β receptor I kinase, Smad2 and Smad3 regulate target gene expression by shuttling from the cytoplasm into the nucleus (Shi and Massagué, 2003). It was interesting to notice that Rb1 was shown to eliminate the HG-induced overexpressions of TGFβ1 and phospho-Smad2/3 *in vitro*, which suggested that the inhibitory effect of Rb1 on the production of HG-mediated collagens may also be involved in TGFβ1. Furthermore, this effect of Rb1 on collagen production could be reversed by compound C, indicating that the effect of Rb1 on the TGFβ1/Smad2/3 pathway was AMPK-dependent.

The findings supported that Rb1 had therapeutic potential in preventing cardiovascular complications in patients with diabetes mellitus, which was independent of decreased glucose levels. Rb1 can reverse the inhibition of AMPK, which, however, may not explain all of its therapeutic effects. Notably, Rb1 was reported to have pleiotropic cardiovascular protection effects on multiple molecular targets independently, mainly including AMPK, PI3K/Akt, NF-κB, and mitogen-activated protein kinase (MAPK) pathways and endoplasmic reticulum stress. AMPK participates in the cardiovascular protection effect of Rb1 against reperfusion injury/myocardial ischemia, coronary atherosclerotic, heart failure, cardiac hypertrophy, and fibrosis by mediating apoptosis (Kong et al., 2010), autophagy (Qiao et al., 2017; Dai et al., 2019), mitochondrial fission (Li et al., 2016), fatty acid β-oxidation (Kong et al., 2018), and aging (Zheng et al., 2020). In the meantime, the changes in Akt signaling are of importance in atherosclerosis, cardiac hypertrophy, vascular remodeling, and many other cardiovascular pathological processes. Rb1 has a cardioprotective effect partly by mediating PI3K pathway activation and Akt phosphorylation and regulating inflammatory response (Yang et al., 2019), oxidative stress (Chen et al., 2019), apoptosis (Nanao-Hamai et al., 2019), autophagy (Yang et al., 2018), and mitochondrial function (Zheng et al., 2017). It was demonstrated that Akt and AMPK pathways in the cardiovascular protection effect of Rb1 were cross and independent of each other. Further studies are necessary to elucidate its integration with other signaling pathways that are predicted to account for this effect.

## Conclusion

Ginsenoside Rb1 ameliorates DM-related vascular remodeling, at least partially, *via* reducing the inhibition of AMPK caused by hyperglycemia. This effect is obtained by alleviating oxidative stress and suppressing TGFβ1/Smad2/3 pathway, leading to regulating collagen production and degradation. Our findings have shown the effect and possible mechanism of Rb1 in treatment for diabetic macroangiopathy and diabetes-related complications prevention.

## Data Availability

The raw data supporting the conclusions of this article will be made available by the authors, without undue reservation.
